# Natural Medicine a Promising Candidate in Combating Microbial Biofilm

**DOI:** 10.3390/antibiotics12020299

**Published:** 2023-02-02

**Authors:** Athar Shamim, Asgar Ali, Zeenat Iqbal, Mohd Aamir Mirza, Mohd Aqil, S. M. Kawish, Ayesha Siddiqui, Vijay Kumar, Punnoth Poonkuzhi Naseef, Abdulkhaliq Ali F. Alshadidi, Mohamed Saheer Kuruniyan

**Affiliations:** 1 Department of Pharmaceutics, School of Pharmaceutical Education and Research, Jamia Hamdard, New Delhi 110062, India; 2 Department of Pharmaceutics, Moulana College of Pharmacy, Perinthalmanna 679321, India; 3 Department of Dental Technology, College of Applied Medical Sciences, King Khalid University, Abha 61421, Saudi Arabia

**Keywords:** biofilm, antibiotics, quorum sensing, natural products, bacterial resistance

## Abstract

Studies on biofilm-related infections are gaining prominence owing to their involvement in most clinical infections and seriously threatening global public health. A biofilm is a natural form of bacterial growth ubiquitous in ecological niches, considered to be a generic survival mechanism adopted by both pathogenic and non-pathogenic microorganisms and entailing heterogeneous cell development within the matrix. In the ecological niche, quorum sensing is a communication channel that is crucial to developing biofilms. Biofilm formation leads to increased resistance to unfavourable ecological effects, comprising resistance to antibiotics and antimicrobial agents. Biofilms are frequently combated with modern conventional medicines such as antibiotics, but at present, they are considered inadequate for the treatment of multi-drug resistance; therefore, it is vital to discover some new antimicrobial agents that can prevent the production and growth of biofilm, in addition to minimizing the side effects of such therapies. In the search for some alternative and safe therapies, natural plant-derived phytomedicines are gaining popularity among the research community. Phytomedicines are natural agents derived from natural plants. These plant-derived agents may include flavonoids, terpenoids, lectins, alkaloids, polypeptides, polyacetylenes, phenolics, and essential oils. Since they are natural agents, they cause minimal side effects, so could be administered with dose flexibility. It is vital to discover some new antimicrobial agents that can control the production and growth of biofilms. This review summarizes and analyzes the efficacy characteristics and corresponding mechanisms of natural-product-based antibiofilm agents, i.e., phytochemicals, biosurfactants, antimicrobial peptides, and their sources, along with their mechanism, quorum sensing signalling pathways, disrupting extracellular matrix adhesion. The review also provides some other strategies to inhibit biofilm-related illness. The prepared list of newly discovered natural antibiofilm agents could help in devising novel strategies for biofilm-associated infections.

## 1. Introduction

Bacterial infections threaten public health worldwide, and the severity of the crisis has intensified due to the appearance and proliferation of drug-resistant bacterial species [1]. The majority of the microbes defend themselves by diverse survival mechanisms, including morphogenesis, proteolytic approaches, demographic heterogeneity, etc., to overcome environmental stress conditions. One of the prominent growth states for microorganisms that exist in 90% or more of bacteria strains is biofilm. Biofilms are considered heterogeneous assemblages of surface-associated microbes compressed in a matrix composed of different polysaccharides, proteins, and DNA. Quorum sensing, an inter and intra-bacterial communication channel, plays a significant role in biofilm development with its extracellular surrounding matrix. Contrary to their planktonic compartments, bacteria residing in biofilm exhibit vulnerable adaptive antibiotic resistance, leading to hindrance in biofilm-associated ill-treatment and chronic infections globally [2,3]. The matrix functions as a physical barrier to medications and provides microorganisms a safe ecological niche for their survival. Many microorganisms, such as *P. aeruginosa*, *C. albicans*, *S. epidermidis*, and *M. tuberculosis Mycoplasma*, pose intense health crises due to antimicrobial medication resistance and immune responses [4].

Consequently, new approaches to combating microbial biofilm formation should be devised. Research suggests that novel approaches to combating biofilm formation and quorum sensing have been widely developed and report that natural drugs show potency in combating drug resistance and antibiofilm illness [5]. Herbal therapies have been used for ages by various human societies; moreover, these natural products play a crucial role in the prophylaxis of infectious diseases. Some Chinese plants, for instance, have frequently been considered in the treatment of bacterial infection, e.g., *Scutellara*, *Tussilago*, etc., [6]. Published articles have shown that different plant extracts regulate biofilm formation and QS inhibition. This article briefly discusses the mechanisms underlying biofilm formation and the fate of quorum sensing along with recent events in the identification of natural products derived from plants that act as antibiofilm agents. The current standard practice for treating bacterial infections is antibiotic therapy. Other strategies include phage therapy, inhibitors of the quorum sensing system, antibodies therapy, antimicrobial peptides therapeutics, smart stimuli-responsive materials, and nanotechnology. These recently identified natural antibiofilm compounds are interesting candidates that may offer fresh approaches to treating infections brought on by biofilm-associated bacteria and pathogenic bacteria.

The application of natural plant-derived ingredients in the field of antimicrobial therapy has emerged as a suitable alternative to combat the growing issues of antibiotic resistance and biofilm formation [7]. These are present in different parts of plants such as the leaf, flowers, stem, root bark, or fruits, which act through different pathways to restrict and inhibit the microbial growth. Some common mechanisms through which they show their effects are: (i) disruption of the microbial cell wall and membrane, (ii) leakage of cellular components as a result of structural alteration, (iii) interference with protein synthesis, (iv) dissipation of ATP, and (v) obstruction in the bacterial quorum sensing. Another benefit of using phyto-constituents as antibacterial and antimicrobial agents are their ease of availability, low cost, and negligible side effects on the body. Thus, sustainable herbal antimicrobial therapy requires further isolation of active plant constituents that could be used as novel antimicrobial agents. Some of the essential phyto-constituents that have already been utilized as antibacterial agents are piperine, dictamnine, berberine, reserpine, conessine, matrine, caffeine, ajoene, baicalein, silybin, quercetin, chrysoplenetin, gallic acid, naringenin, toxifolin, limonene, nerolidol, linalool, etc. [8]. These ingredients follow different pathways, the details of which have been mentioned in the text.

## 2. Biofilm

Biofilm is a congregation of microorganisms where planktonic cells cling to a surface [9]. These aggregated colonies are usually sheathed in a self-produced extracellular polymeric substance (EPS), a matrix cell sometimes known as slime (though not all slime consider biofilm), a polymeric aggregation comprised primarily of various proteins, extracellular saccharides, and DNAs [10,11]. Considering the composition of the EPS matrix, biofilm may evolve into a variety of distinct forms, including filamentous streamers and microcolonies that resemble mushrooms [12]. It has been established that the morphology of biofilms in microcolonies is regulated by fluid flow, nutritional makeup, and the messenger molecules called quoromones. N-Acyl homoserine lactones (AHLs) are utilized for bacterial communication. [13,14]. Biofilms can evolve on non-living and living surfaces and are frequently seen in the natural world and in healthcare facilities. In contrast to planktonic cells, single cells of the same organism that grow in biofilms show different physiological properties [15]. Research has shown that the microbial cells in the biofilms hold the ability to mutate, which is the leading cause of developing resistance to antibiotics used in eroding the biofilm [16,17]. Each and every component plays a critical role in biofilm structure; that is, Pel and psl polysaccharide helps provide a solid surface for biofilm, whereas the alginate provides the surface adhesion. In case of starvation, eDNA functions as a nutrient source [18,19].

### 2.1. Stages of Biofilm Formation

**Figure 1:**
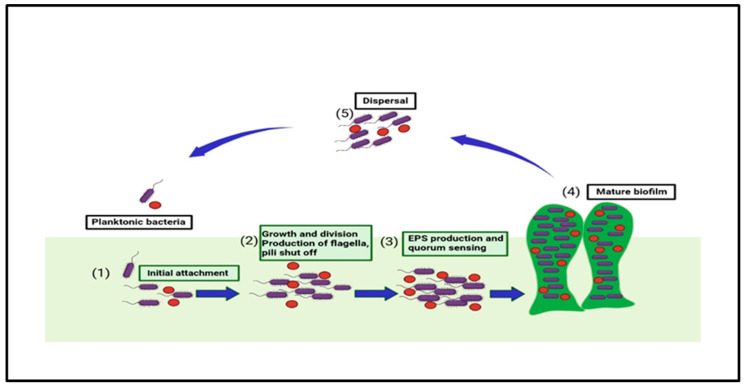
This figure shows the five stages in biofilm formation and the associated factors that take part in biofilm development and quorum sensing.

There are five steps found in forming biofilm, as shown in Figure 1. They start from the initial stage of attachment of planktonic microorganisms to a surface. The initial stage (reversible) is defined as a set of physiochemical factors that specify how the bacteria’s cell surfaces interact with the prepared surface [20]. The secondary stage consists of the specific adhesion with the surface; the bacteria in this stage undergo both phenotypic and genotypic changes. The third stage is maturation, wherein the irreversibly attached bacteria communicate with the nearby organic and inorganic molecules [21]. Microcolonization of the bacteria can be defined as the fourth stage in which the association also becomes stable, and the bacteria start the step of intercommunication among different bacteria [22,23]. The final fifth stage is dispersion, where factors such as fluid dynamic and shear stress cause microbial dispersal from the older biofilm; thus, either shedding the daughter cells and causing the microorganisms to move to the newer locations or leading to the formation of other biofilms [24,25].

### 2.2. Mechanism of Biofilm Formation

**Figure 2:**
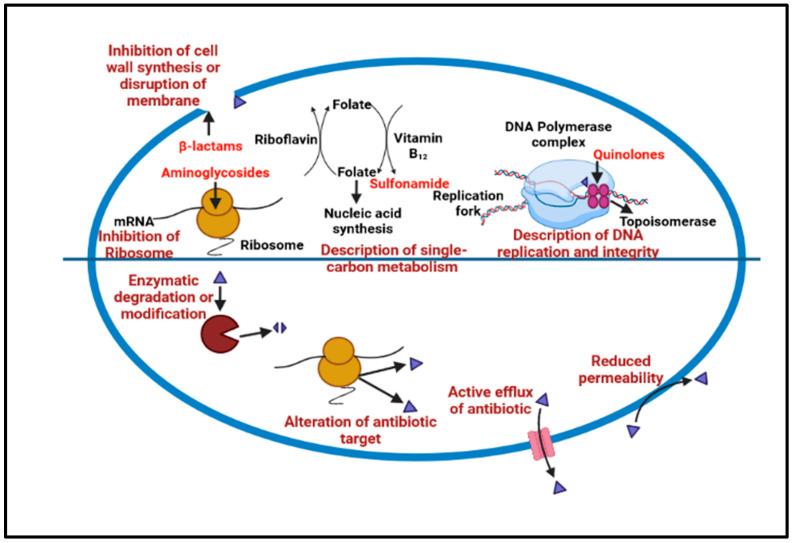
Show the common antibiotic resistance mechanism that leads to biofilm development.

The microbial cells in the biofilm are composed of extracellular polymeric substances (EPS) with a larger molecular weight, causing resistance to many acids as well as metals; as well as this, the other causes of this resistance are a genetic adaptation, cell growth, and metabolic changes [26]. The mechanisms of biofilm tolerance can be explained as the synthesis of EPS, prompting physical tolerance, leading to difficulty in antimicrobial diffusion inside the matrix. The second type of tolerance caused is passive tolerance, which is basically due to the enzymes of the biofilm matrix that cause the antimicrobial agents’ neutralization [27]. The third one is physiological tolerance, in which the deeper layers of the biofilm have metabolically dormant cells that demonstrate adaptive stress responses that modulate biofilm resistance to different antimicrobials [28]. During biofilm maturation, the microbial cells inside the EPS begin to communicate and secrete specific proteins, which are responsible for efflux pump development. Finally, free planktonic cell dispersal from the produced biofilm encourages the creation of additional biofilms in the periphery [29]. Many naturally obtained biofilm-resistant drugs target various stages of biofilm production and inhibit growth. Specific strategies could be targeted to degrade the cells in the matrix to halt the biofilm mechanism that inhibits QS, preventing efflux pump expression, and inhibiting matrix formation and surface modification, as shown in Figure 2.

### 2.3. Quorum Sensing and Its Association with Biofilm Development

**Figure 3:**
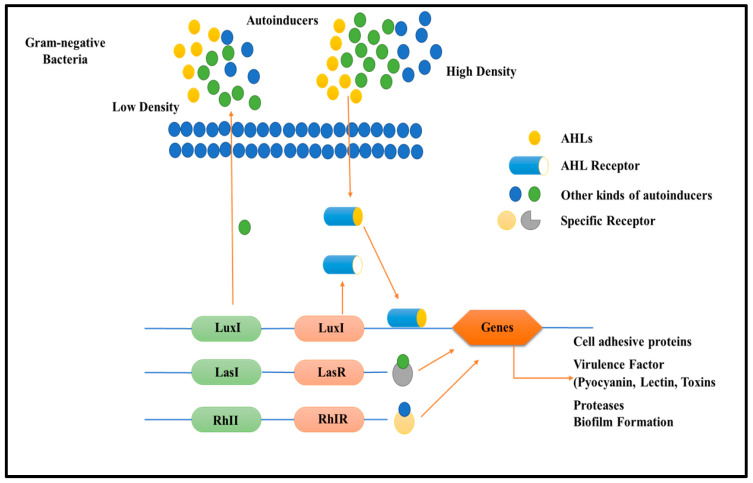
Diagram describes quorum sensing signalling mechanism in Gram-negative bacteria relation and biofilm development role. Gram-negative bacteria frequently created (AHLs) during communication, which activated the relevant cytoplasmic receptors to modify the expression of the targeted genes. *Luxl/luxR* transcriptional factors of QS in Gram-negative bacteria.

**Figure 4:**
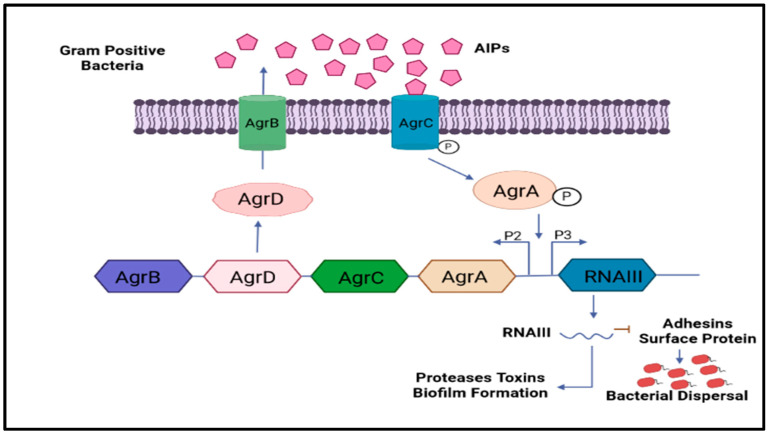
Shows the involvement of Gram-positive bacteria’s classical QS signalling molecules and their role in biofilm development. The common QS signalling system is Agr.

Multi-drug resistance is mainly caused by the rigid development of biofilm, which is a complex structure developed in four to five different stages, starting with the irreversible attachment (initial) of planktonic bacteria, the second step is colony formation, the third one is bacterial growth, the fourth is extracellular matrix generation, and, finally, the biofilm’s maturation leading to the final reversible detachment of cells from the older formed biofilm [30,31], see Figure 1. The exopolysaccharide or (EPS) is composed of so many essential nutrients among which Aap protein associated with the cell surface contains the G-5 domain, accountable for cell adhesion; during infection in Gram-negative time bacteria (Figure 3), *Sortase A (SrtA)*, a transpeptidase, induces extracellular localization and biofilm generation and can bind cell surface proteins [32,33]. Thus, it can be concluded that inhibitors against this adhesion can potentiate the antibiofilm activity [34]. The communication among the cells is dependent on a chemical signal molecule termed as quorum sensing (QS) [35]. The QS mechanism varies between bacteria, as evidenced by the production of signalling molecules in Gram-negative bacteria N-acyl-homoserine lactones (AHLs) as described in Figure 3, whereas in Gram-positive bacteria, the signalling molecule is peptides (autoinducer 2), which are described in Figure 4. Thus, similar to the adhesion inhibitors discussed above, some other enzyme shows the enzymatic degradation of QS signals caused by different enzymes such as lactonase, various acylase, and paraoxonase oxidoreductase, and the enzyme may provide a promising strategy for regulating biofilm development. As a result, the inhibition of QS and biofilm development has been reported in numerous studies [36,37,38]. It has been observed that many of the natural QSIs show a remarkable degradation of biofilm when combined with antibiotics [39], for example, usnic acid, which is a derived metabolite of lichen origin, and interfere with QS resulting in a changed morphology of *S. aureus* as well as of *P. aeruginosa* [36,40]. The biofilm has a specific relevance in terms of the QS system for a specific gene expression as well as communication among the cells; pyocyanin is a QS-mediated product helpful in the maturation of biofilm by interacting with eDNA released after the cell lysis and causing a viscous solution and thus aggregation of the cells and the production of an established mature biofilm [41,42]. In most of the microbes, the quorum signalling system, viz, *rhl*, *iqs*, *pqs*, *and las*. *P. aeruginosa las and rhl* are QS, is an expression for virulence genes. The *las* and *rhl* are further divisions in which *LasR*, acts as a transcriptional activator protein and is associated with AHL synthase *LasI*, which controls the production autoinducer, N-(3-oxododecanoyl)-l-homoserine lactone [43,44]. Likewise, the N-butyryl-l-homoserine lactone (AHL) of the Rhl system, which *RhlI* produces, is composed of the transcriptional activator *RhlR* [45].

As a result of the ongoing drug resistance, it is essential to search for alternative antimicrobial medications. It has been observed that the *las* and *rhl* QS systems use either a direct or indirect method to regulate their expression of environmental changes. Quorum quenching is one of these, and it impairs microbial quorum sensing. It minimises the virulence factors’ synthesis and biofilm formation. Only a few quorum quenching (QQ) methods use structurally similar QS receptor autoinducers (AI). They are natural or synthetic chemicals. Most of the QQ molecules that have been discovered are enzymes that can degrade signalling molecules, preventing the creation of biofilms [31,46]. Thus it can be concluded that quorum sensing inhibitors, whether from a natural or synthetic origin, when combined with antibiotics, can help erode biofilms [47,48,49].

## 3. Natural Product as Antibiofilm Agent

**Figure 5:**
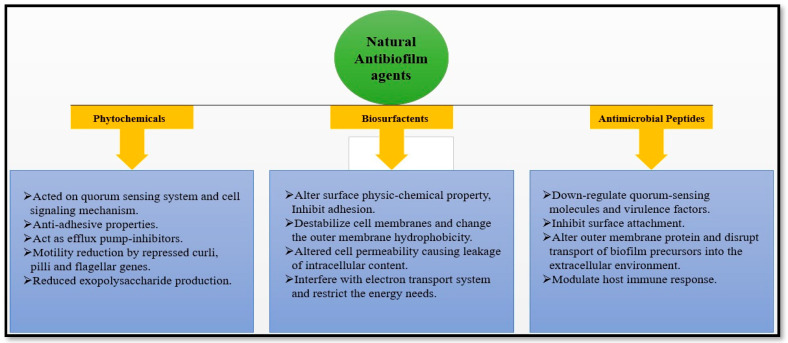
Natural antibiofilm agents based on their mode of action.

Natural medicines have been used for centuries. Various plant-derived natural compounds exhibit in vitro antimicrobial and antibiofilm properties [50]. Numerous plant-derived molecules or medicinal herbs, and natural compounds show remarkable prophylaxis effects for bacterial biofilm infections, and their mechanisms of action in antibiofilm activity have been identified [7,51]. The development of biofilm is a very complex process. The factors responsible for the antibiofilm activity of natural drugs prevent polymer matrix development, inhibit cell adhesion and attachment, prevent the formation of extracellular matrix (ECM), and limit the virulence factors’ production, thus leading to the development of the QS system, and the biofilm is restrained. Targeting one or both of the initial two stages crucial for developing biofilms seems to be the best action to prevent their creation [52]. Antibiofilm activity of the natural product is divided into three subcategories based on their mode of action, as shown in Figure 5 and Table 1; the potential antibiofilm activity of a few natural antimicrobial drugs is shown.

### 3.1. Phytochemicals

Plants produce substances that may not be essential for their primary metabolism but exhibit their potential to adapt to harsh abiotic and biotic environmental conditions. Extensive research was conducted on various plant extracts and their active ingredients to get rid of the *Propionibacterium acne* biofilm [53,54]. Studies showed that these extracts (*Dolichos lablab*, *Polygonum cuspidatum*, *Malus pumila*, *Rhodiola crenulata*, *and Epimedium brevicornum*) have significant antibiofilm activity [55]. The research also revealed that *P. cuspidatum* and *E. brevicornum* extracts with icartin and resveratrol as active components demonstrated considerable antibiofilm activity below their minimal inhibitory concentrations [56]. After being tested, *Melia dubia* bark extracts effectively regulate *E. coli* biofilm development [57,58,59] Extract from the caper bush *(Capparis spinosa*) effectively reduced the growth of biofilms and the production of EPS in *S. marcescens*, *E. coli*, *P. aeruginosa*, *and P. mirabilis* [60,61]. Southeast Asian native herb *Lagerstroemia speciosa* significantly reduced biofilm growth in *P. aeruginosa PAO1* [62,63]. The aqueous extracts of *Brazilian xeric* show antibiofilm activity and serve as an *in vitro* screening of *S. epidermidis* [64]. According to the study, biofilm development was significantly decreased by extracts of *Bauhinia acuruana*, *Chamaecrista desvauxii*, and *Pityrocarpa moniliformis*. Moreover, *Senna macranthera* and *Commiphora leptophloes* fruit extracts were said to diminish biofilms by 67.3% and 66.7%, respectively [65]. Using qualitative and quantitative methods, a study on the growth of *M. smegmatis* biofilm revealed the role of various herbs, spices, and plants, including *Vaccinium oxycoccus*, *Azadirachta indica*, *Juglans regia*, and *Hippophae rhamnoides*. The extract of *A. indica* was shown to be the most effective on *M. smegmatis* biofilms [66,67].

Natural substances with strong antibiofilm characteristics fall roughly into eight types, flavonoids, terpenoids, lectins, alkaloids, polypeptides, polyacetylenes and phenolics, and essential oils [68]. There are seven subclasses of phenols, and condensed tannins, which mainly show antibiofilm activity [64]. These compounds act on biofilms using different mechanisms, including substrate restriction, cell wall rupture, acting on adhesion groups and cellular membranes, protein binding, and DNA association, leading to blocking viral fusion [69,70]. According to a published research article, antibiofilm capabilities of Indian-originated herbs, *Bischofia javanica*, *Syzygium* Roxb, Rathakr. *Mazz*, and *Cinnamomum glaucescens* (Nees) showed positive results. Extract of *Holigarna caustica* (Dennst.), extract of gum Arabic tree *Acacia nilotica* (L.), Anacardiaceae family, extract of jack of orange jasmine *Murraya paniculata* (L.), and fruit extract of Buddha Coconut *Pterygota alata*, act on *S. aureus* against their biofilm development [71]. *Salvia officinalis* L., a medicinal herb from Algeria that contains 12-Methoxy-trans-carnosic acid and abietane diterpenoid, also known as carnosol, demonstrated antibiofilm efficacy against *Candida* [72]. Phytochemicals generally act on the inducers molecule such as AHL and may act on some other autoinducer and type 2 receptors, to stop the functioning of the QS signalling system [73]. Garlic extracts exhibited a significant inhibitory role in the QS signalling compound of *P. aeruginosa* biofilm and other species’ biofilms such as *Vibrio spp*. [6,74]. Emodin is a potent inhibitor and facilitates the protein degradation of transcription factors involved in quorum sensing [75].

Numerous researchers have shown that quorum quenchers and antibiotics combination might be considered as the best non-traditional antibiofilm agents [46]. Furthermore, phytochemicals are essential for suppressing the genes that lead to biofilms forming and preventing bacterial adhesions [76]. Biofilms in their early phases can be disturbed by the fluid dynamic properties of the planktonic cell, an electrostatic force of attraction, and properties of sedimentation that encourage attachment to diverse surfaces [34]. Phytocompounds have the ability to stop the availability of essential nutrients required for bacterial attachment and growth. The *Psidium guajava* L. extracts in ethanol and acetone, as well as extracts from several Eugenia species, have been shown to have antiadhesive effects on *C. albicans* [77]. *Syzygium aromaticum* (*S. aromaticum*) extract shows antibacterial activity in dental caries against various strains of *streptococci* [78]

Norbgugaine significantly impacted *P. aeruginosa* biofilm by inhibiting cell motility caused by an inability to adhere to the surface [79]. *Adiantum philippense* L. crude extract has the potential to reduce the number of exopolysaccharides in biofilms [76]. They discovered that crude extract of *Adiantum philippense* L. blocks the generation of EPS, targets adhesin proteins, and deforms already-formed biofilms to prevent biofilm growth in the initial stages. In terms of different phytocompounds, numerous studies have discovered that polyphenols, particularly those such as 7-epiclusianone (tetraprenyl benzophenone derivative), casbane parent hydride of casbene, and tannic acid, a type of polyphenol, inhibit surface attachment [76,80,81]. *Enterobacteriaceae’s curli*, an amyloid fibre expressed on the EPS surface, encourages the formation of biofilms, cell aggregation, and target attachment [82,83] They revealed that the flagellar operon *flhDC* was suppressed by the citrus sterol-sitosterol glucoside, which prevented the production of biofilms and cell motility in *E. coli* O157:H7. Curlicide derivative (Aβ-peptide) and pilicide phytocompounds are used as new therapeutic approaches to prevent *Enterobacteriaceae* biofilms [84]. Pyridones interfere with curli, leading to expression modification and biogenesis of the CsgA. (*Curli*, *pilli*) genes are regulated by phytochemicals from Malaysian plants, including phloretin, ginkgolic acid, and phytocompounds [84,85,86].

Garlic extract has been shown to inhibit QS in the research of Bjarnsholt. It was found that the administration of garlic extract for a lung infection (mouse model) reduces the drug resistance of tobramycin and the neutrophils (PMN) phagocytosis of *P. aeruginosa* [87]. Garlic extract diminishes QS signals, and virulent factors of *P. aeruginosa* are reported in the UTI model [88]. Six clinical bacterial isolates showed resistance to the biofilm-forming activities of garlic extracts [89,90]. Plant extracts from *Coptis chinensis* and *C. trilobus* may prevent germs from adhering to fibronectin-coated surfaces. They show the effects at the adhesion stage by inhibiting sortase (an enzyme of biofilm development) and enhanced the covalent attachment of surface proteins in Gram-positive bacteria [91,92]. The polyphenols found in cranberries prevent the growth of biofilms and the colonisation of pathogens [93,94]. Cranberry components exhibited promising effects for the deterrence and cure of oral infections, such as dental caries and periodontitis, by impacting binding proteins (glucan); the enzymes acted by splitting down the ECM, energy production, hydrophobicity of the biofilm, and degradation activities [95]. According to studies, *Ginkgo biloba* extract significantly reduced the development of fimbriae and biofilm in *Escherichia coli* O157:H7; this agreed with the inhibited *curli* and prophage genes [96,97]. *E. coli’s* ability to swim was found to be impaired by cinnamaldehyde, which also allegedly affects biofilm formation and structure [98]. Citrus limonoids are distinct secondary triterpenoid metabolites.

The purified limonoids demonstrate their capacity to obstruct *Vibrio* harve’s cell–cell communication and biofilm development. Bacterial cell–cell signalling is effectively modified by isolimonic acid [98,99], ichangin block biofilm, and the type III system. Additionally, it disrupts the AI-3, which triggers the signalling pathway according to QseBC and QseA dependence [100]. Hordenine’s anti-QS potential is a signalling molecule competitor inhibitor and novel for foodborne infections. Hordenine successfully decreased the QS-related gene expression *in P. aeruginosa* PAO1 [101,102,103]. Hordenine-conjugated AuNPs showed improved antibiofilm properties on *P. aeruginosa* PAO1 [104]. It has been discovered that different gene levels (*LasI*, *LasR*, *RhlI*, *and RhlR*) for QS signalling are significantly lowered by the presence of the plant polyphenol quercetin [105]. Quercetin potently inhibits *P. aeruginosa* pathogenicity and biofilm formation [106]. It is a potent SrtA antagonist that could significantly prevent the formation of *S. pneumoniae* biofilms, causing the inhibition of sialic acid expression [107]. *Streptococcus mutans* and *Enterococcus faecalis* antibiofilm activities of quercetin in biofilm production, and biofilm-related infections were also explored, and the results indicated quercetin’s potential for application in human health bacterial infection and anticaries therapy [108,109]. Natural antibiofilm agents’ screening has expanded in scope over time. Other antibiofilm substances, such as those made from herbs, are, in addition to those mentioned [110], medicinal plants, phenolic compounds [111], green tea [112], mushrooms [113], licorice root [114], *Polish propolis* [115], *Allium sativum* [116], *Psidium cattleianum* leaf [116], *Solidago virgaurea* [117], *Roselle calyx* [118], and *Juglans regia* L. [119].

#### 3.1.1. Essential Oils

Essential oils (EOs) are aromatic compounds that plants produce; they are nonbiocidal and show promise as a treatment for bacterial biofilm illness and increased drug resistance in vitro [120,121]. In the case of *S. epidermidis* strains, EOs primarily function as biofilm inhibitors. Results have demonstrated that they can destabilise biofilms at deficient concentrations without compromising bacterial viability due to their effectiveness against *P. aeruginosa*. The effectiveness of cumin oil in contradiction of the biofilm production by *pneumonia* strains was investigated [122]. This study showed that this EO decreased biofilm growth and improved the antibiotic ciprofloxacin’s effectiveness. The research reveals that cinnamon oil is effective against the biofilm of *S. mutans* and *Lactobacillus plantarum* [123,124]. Cinnamon oil effectiveness was reported in *S. epidermidis* biofilm [125]. The Enteropathogenic *E. coli* attachment stage in biofilm is suppressed by Cinnamomum oil [126]. The essential oil derived from oregano showed disruptive biofilm properties of *staphylococci* and *E. coli* species [127]. Oregano EO exerts antibacterial potency on different species of *Staphylococcus* and *E. coli* planktonics, and prevents biofilm formation [127,128]. Recently, studies have been conducted on the antibiofilm properties of vegetable oil (Brazil nut oil) on dentifrice biofilm [129]. It was claimed that using commercially available dentifrice with vegetable oil could help prevent and treat dental caries and periodontal disease because of its antibacterial properties. With regards to tea tree oil, the tea tree EO (TTO) and ciprofloxacin (CIP) combination and their antibacterial activity were evaluated on *P. aeruginosa*. The result revealed that the synergistic effect of the combination reduces the biofilm biomass by 70%. According to reports, EOs (thymol, cinnamon oils and oregano) were found to be effective against the biofilm-forming strains of *Acinetobacter*, *Sphingomonas*, and *Stenotrophomonas* at sublethal concentrations [130,131].

#### 3.1.2. *Andrographis paniculata* (Andrographolide)

*Andrographis paniculata* (*A. paniculata*), commonly found in China, belong to the *Acanthaceae* family [132]. It has proven successful in treating bacterial infections and has a sizable impact *on P. aeruginosa*, *S. aureus*, *and E. coli* biofilm generation [133,134] and decreases the synthesis of extracellular pathogenic factors in *P. aeruginosa* regulated by the QS system, such as anthocyanins and elastase. Andrographolide (AG) is the active metabolite of *A. paniculata;* AG inhibits biofilm formation by inhibiting the QS system by regulating *SarA* factor, which is responsible for biofilm development in *S. aureus* [135]. *E. coli* adhesion can be reduced by AG as the amount of PIA/PNAG declines because this also affects the ability to produce biofilms [136].

#### 3.1.3. *Polygonum cuspidatum* (Emodin)

*Polygonum cuspidatum’s* constituents are anthraquinone, emodin, etc. [137]. One goal of caries prevention is to control dental plaque. Since biofilm formation in the dental cavity starts with the salivary membrane and planktonic bacteria adhering to the surface of teeth, reduced planktonic bacterial loads may help regulate biofilm development [138].

#### 3.1.4. *Curcuma longa* (Curcumin)

*Curcuma longa* of the Zingiberaceae family produces sesquiterpenes, turpentine, and fatty acids that disrupt antibiofilm activity, impairing the bacterial cell membrane’s normal barrier function, and is crucial for the growth and metabolism of bacteria [139]. It modulates various QS-dependent pathogenic factors, such as *Vibrio spp*. swarming, alginate synthesis, and mobility [140,141]. It works by blocking the growth of EPS and decreasing the flagellum effect, which restricts bacteria from swimming and the development of biofilms [140,142].

#### 3.1.5. *Allium sativum* (Allicin)

Ajoene exhibits QS inhibitory activity in *Allium sativum*, where organic sulphide is the main antibacterial component and decreases the *rhlA* gene in *P. aeruginosa*, lowering the concentration of rhamnolipids encrypted by *rhlA*. Garlic extract inhibits the biofilm formation in *L. monocytogenes* and *P. aeruginosa* [116], and garlic checks the virulence factors and QS signal generation in *P. aeruginosa* [88].

**Table 1:** The potential antibiofilm activity of a few natural antimicrobials drugs.

Plant Extract/Compounds	Botanical Source	Species	Mechanism	Antibiofilm Effects	References
L-homoserine lactone (Garlic extract)	*Allium sativum* L.	*Pseudomonas aeruginosa*	Acted on transcriptional pathway (*LuxR* and *LasR*)	Reduced synthesis of QS signals and decreased pathogenicity.	[61]
Carvacrol (terpenoid)	*Origanum vulgare* L.	*Pseudomonas aeruginosa*	Inhibition of *lasI*, which affects AHL production.	It acts by post-translational inhibition enzyme and interrupts the QS signaling mechanism	[143]
Polyphenols (cranberry)	*Vaccinium oxycoccos*	Cariogenic and periodontopathogenic bacteria	Glucan-binding proteins, enzymes used in biofilm formation	Impaired biofilm development by coaggregation, degradation of ECM, glucose synthesis, bacterial hydrophobicity, and photolytic activities.	[65]
Ajoene	*Allium sativum* L.	*P. aeruginosa*, *S.aureus*	Impedes RNA regulatory molecules (rsmY, *rsmZ*, and *rnaIII*) and reduces rhamnolipid synthesis	Limits the QS signalling RNA regulatory molecules	[144,145]
Emodin (anthraquinone)	*Origanum vulgare* L.	*Staphylococcus aureus*	Reduces eDNA production, and impairs gene regulator (cidA, *icaA*, *dltB*, sortase and AagrA)	It regulates eDNA, and inhibition of gene expression, which is essential in biofilm formation (*cidA*, *icaA*, *dltB*, *agrA*, *sortaseA*, and *sarA).*	[146]
Allicin	*Allium sativum* L.	*Pseudomonas aeruginosa*	Inhibits EPS production, regulating the pathogenic factors.	Inhibits EPS production, reduces bacterial adherence at the primary stages of biofilm generation, and acts on the QS system by altering the expression of virulence factors.	[147]
Hordenine	*Hordeum vulgare* L.	*Pseudomonas aeruginosa*	Impaired AHL production, gene regulation *rhlR*, *rhlI*, *lasI*, and *lasR* genes.	Limits the generation of AHL resulting in bacterial biofilm generation	[101]
Vitexin (flavonoid)	*Vitex species*	*P. aeruginosa*	Inhibit EPS, by acting on *LasA*, *Las B*, and *Lux R*	Reduces the production of proteolysis enzyme, and surface protein as well as EPS and components linked with QS. Attenuates *LasA*, *Las B*, and *Lux R*	[148]
Patriniae	*Patrinia scabiosifolia*	*Pseudomonas aeruginosa*	Acts on biofilm-associated genes	Decreased exopolysaccharide synthesis and prevented biofilm development	[67]
Ginkgolic acids (Leaf extract)	*Ginkgo biloba*	*E. coli* O157:H7	Acted on Curli and prophage genes	Prevented biofilms’ production on non-living surfaces such as nylon, polystyrene, and glass	[149]
Phloretin (Natural phenol)	*Annurca apples*	*S. aureus RN4220 and SA1199B*	Efflux protein genes	Production of an antibiofilm at low concentrations	[58]
Cinnamaldehyde (Phenylpropanoid)	*Cinnamomum ceylanicum*	*E. coli and Vibrio spp.*	DNA-binding ability of *LuxR*	Inhibits biofilm development by regulating structure, flagella of bacteria, and stress responses	[121]
Phloretin (Natural phenol)	*Annurca apples*	*E. coli O157:H7*	It acted on Curli genes (*csgA* and *csgB*), toxin genes (stx(1)), autoinducer-2 importer genes (DE3),	Inhibited biofilm formation and fimbria production	[58]
Emodin (Anthroquinone)	*Polygonum cuspidatum* *Siebold & Zucc.Rheumpalmatum* L.	*Candida spec*. like *C. albicans*, *C.krusei*, *etc.*	Acts on cellular kinase signalling and CK2	Inhibits biofilm development by acting on cellular kinase signalling. Disrupts planktonic cells by inhibiting the growth of hyphae.	[131]
Pulverulentone A (Skeels leaves)	*Callistemon citrinu*	Methicillin-resistant *S. aureus*	Inhibits staphyloxanthin production	It decreases staphyloxanthin synthesis, and the cell membrane is impaired, preventing biofilm growth.	[150]
Tannic acid	*not defined*	*E. coli* BW25113	Prevents the generation of polysaccharides in the matrix.	Impeding generation of saccharide in ECM. Regulates SOS cell to cell response and declines to kill bacteria in pgaA mutant biofilms.	[151]
Aloe-emodin	*Rheum officinale Baill.*	*Staphylococcus aureus*	Inhibit extracellular proteins of the matrix	Limits ECM extracellular polysaccharide and protein adhesion	[152]
5-Hydroxymethylfurfural	*Musa acuminata Colla.*	*Pseudomonas aeruginosa*	Prevents the synthesis of EPS, cell protein	Prevents the synthesis of EPS, and extracellular proteins, limits hydrophobicity, regulates expression of pathogenic genes that QS controls	[153]
Isolimonic acid and ichangin	*Citrus species*	*Enterohaemorrhagic*, *E. coli*, *Vibrio harveyi*	Inhibits gene regulator for flagella flagellar (*flhC* and *flhD*), *luxO* expression	Diminishes adherence, reduces the expression of ler (transcriptional regulator of LEE), a gene involved in making the flagellum. Suppresses master regulator’s expression (*flhC* and *flhD*) and controls *luxO* expression, effectively modifying bacterial cell–cell signalling.	[154]
Chelerythrine	*Bocconia cordata* willd.	*Candida albicans*, *Staphylococcus aureus*	Acts on hyphae formation, eDNA, regulation, inhibit saccharide, and protein levels	Hinders the development of hyphae, decreases the quantities of eDNA, polysaccharides, and proteins to reduce the production of biofilms.	[155]
Quercetin	*different sources*	*Streptococcus Pneumoniae*, *Pseudomonas aeruginosa*	*SrtA*, *LasI*, *LasR*, *RhlI* and *RhlR*	It acts by blocking SrtA, gene which checks the sialic acid generation and inhibit biofilm formation	[74,75,76,77]
Methanolic fraction of Zingiber officinale	*Zingiber officinale*	*S. mutans*	Impairs protein generation factor F-ATPase, surface protein antigen SpaP	It regulates surface protein by impairing antigen SpaP, showing effect on cell surface hydrophobicity in *S. mutans*	[64]
Methanolic caper bush extract	*Capparis spinosa* L.	*S. marcescens*, *E. coli*, *P. aeruginosa*, *and P. mirabilis*	Synthesis of EPS producing enzyme	Effectively reduced the growth of biofilms and the production of EPS	[64]
Emodin	*Rheum palmatum*	*Pseudomonas and Vibrio spp.*	Transcription factors in QS	Potent inhibitor and facilitates degradation of transcription factors involved in quorum sensing	[156]
Guava alcoholic extract	*Psidium guajava* L	*C. albicans*	Inhibits cell motility enzyme.	Inhibits primary-stage adhesion due to a lack of motility, impacted on biofilm development	[66]
12-Methoxy-trans-carnosic acid and carnosol	*Salvia officinalis* L	*Candida*	QS sensor disruption (AHL), autoinducers, and type 2 receptors	Inhibits biofilm formation using quorum sensing system from functioning by QS disruption sensor inducers (AHL), autoinducers, and type 2 receptors	[64,65]
Norbgugaine	*Arisarum vulgare*	*Pseudomonas aeruginosa*	Acts on cell motility proteins	Inhibits a primary attachment caused by cell motility, showed significant impact on biofilm formation	[52]
Curlicide and pilicide	*not defined*	*Enterobacteriaceae*	Acts on the flagellar operon *flhDC* enzyme	Inhibits the cell motility by blocking flagellar operon *flhDC* enzyme production biofilm development	[57]

### 3.2. Biosurfactants

Biosurfactants (BS) impede the capacity of cells to attach to surfaces by affecting the hydrophilic nature of the cell membrane, perforating membranes, and limiting the electron transport chain, thus decreasing the energy need of cells [157]. Medical implants such as urinal catheters and bone implants can be coated with biosurfactants to prevent biofilms from harmful organisms without synthetic medications [158]. *Staphylococcus aureus* was tested for how biosurfactants from *Lactobacillus Plantarum and Pediococcus acidilactici* affected the expression of biofilm-related genes and quorum sensing signalling molecules [146,159]. As claimed from the study, biosurfactants control gene expression related to biofilms, such as *dltB*, *icaA*, *cidA*, etc., which control the development of *S. aureus* biofilm [158]. At 50 mg/mL, *Pediococcus acidilactici* biosurfactant inhibits gene production such as (AI-2) signalling factor and *staphylococcal* accessory regulatory-*sar A* [158]. According to earlier research, BS-loaded liposomes made from *Lactobacillus* were more efficient than free BS, preventing the development and removal of *S. aureus* (MRSA) biofilms [160,161]. According to earlier research, BS-loaded liposomes produced from *Lactobacillus* were more efficacious than plain BS at preventing the development and removal of S. aureus (MRSA) biofilms. Table 2 summarizes some biosurfactants with their effect on biofilm development.

The lipopeptide component of the anionic bacteria *Acinetobacter junii* is found to self-assemble and create vesicles filled with biosurfactant sheets. A lipidic biosurfactant of the fungus *Beauveria bassiana* is essential for *M. canis* to function as a biofilm inhibitory agent in ex vivo settings [162]. It impacted membrane permeability and started to break down the integrity of the cell membrane. Cyclical metal complexes were discovered to be highly efficient against infections brought on by *C. albicans* biofilms [163]. Surfactin does not have the same antiadhesive and antibiofilm properties as *Pseudomonas aeruginosa* MN1 rhamnolipids [164]. The glycolipid produced by *Burkholderia sp. WYAT7* showed ant biofilm activity against *Staphylococcus aureus* [165] as an endophyte of *Artemisia nilagirica* (Clarke) Pamp [166]. *Burkholderia cepacia* is resistant to biofilm formation *Burkholderia pnomenusa* MS5 [167]. According to reports, rhamnolipids and sophorolipids may be effective inhibitors of the biofilms developed by all classes of microorganisms [168]. Several studies have demonstrated that when exposed to cell-associated biosurfactants from *Lactobacillus acidophilus*, *S. aureus*, and *Proteus vulgaris* cannot develop biofilms on (PDMS)-based implants [169]. Biosurfactants from *L. rhamnosus* promote cell lysis by upsetting the structure of the membrane, making them an effective antibiofilm agent for diverse microbial biofilms on silicone implants, such as voice prostheses in *laryngectomy situations* [170]. Caprylic acid, which prevents the growth of biofilms from *P. aeruginosa*, *E. coli*, *and B. subtilis*, can significantly increase the antibiofilm efficacy of biosurfactants [171]. Fluconazole and (AmB) work together to prevent the growth of a biofilm and the existing biofilm of *Candida albicans* [172]. Surfactants such as sodium dodecyl sulfate caused *P. aeruginosa* PAO1 biofilm’s inhibition. [173].

### 3.3. Antimicrobial Peptides

Broad-acting antimicrobial medications (AMPs) are commonly used against bacterial and fungal biofilms. Infections caused by *S. aureus*, *K. pneumoniae*, *P. aeruginosa*, *Acinetobacter*, and *Enterobacter spp*. (ESKAPE) as well as non-ESKAPE microorganisms are interrupted by these peptides acting on medical specimens such as valves, stents, and dentures [174,175]. Antimicrobial AMPs can block various biofilm formation molecular pathways [176]. The several AMPs in amphibian skin are efficient against different microbes that cause biofilms. AMP *Japonicin-2LF*, which inhibits MRSA biofilms by permeabilizing membranes, is a derived secretion from the skin of *Limnonectes fujianensis*, a frog from Fujian. Both sessile and planktonic pathogens are eliminated from biofilms by the detergent-like behaviour of *Japonicin-2LF*. Esculentin-1a, also known as Esc, is an AMP obtained from frog skin; its Esc contains D-amino acids (1–21). By disrupting membranes, -1c inhibited the biofilms of *P. aeruginosa* [172]. Three processes work together to prevent *P. aeruginosa* from forming biofilms. The *fleN* gene, which determines the flagella number in *P. aeruginosa*, is first downregulated, which prevents flagella-mediated swimming. Second, it inhibits *P. aeruginosa* twitching motility, which is crucial for microcolony formation and colonization during biofilm growth. It reduces type IV pili synthesis genes’ mRNA levels at a low concentration. Third, it prohibits the *lasB* gene, which codes for the virulence factor elastase LasB, and the *lasI* gene, which codes for the QS protein (AHL) synthase. It also carries out its function by electrostatically binding to the negatively charged bacterial cytoplasmic membrane, causing the creation of pores as a result of the solubilization of the membrane, which results in its destruction [177]. Due to their unique qualities of limitless sequence space and ability to produce antimicrobial action, these AMPs become the perfect candidate for overcoming microbial resistance. Additionally, some low molecular weight AMPs that penetrate the cellular structure act by becoming bound to the microbial genetic material, triggering the ROS-dependent pathway for their antimicrobial effect. The AMPs are mainly classified as defensins and bacteriocins. The defensins are the class of AMPs and peptides obtained from natural sources. This class of AMPs is also known as HDPs (host defence peptides), which act as broad-spectrum antibiotics and perform their action by electrostatic interaction at the microbial membrane, causing membrane destruction and causing a bactericidal effect [178,179]. On the other hand, bacteriocins are peptides obtained from bacteria, which are synthesized in the ribosomes. The first class of bacteriocins were discovered in 1925, after which a number of different forms of bacteriocins were discovered [180]. Although functionally they also perform antimicrobial and antibiofilm activities very similar to that of defensin, they are different from defensin in that this cationic amphiphilic molecule, in addition to becoming attached to the bacterial cell, causes the destruction of the phospholipid membrane, and hence obtains access to the DNA component and organelles of the cell, causing direct interference with the protein synthesis process. Thus, both forms of AMPs have proven to be valuable weapons in dealing with resistant microbes, which usually avoid killing via biofilm formation.

## 4. Other Ways to Inhibit Biofilm Resistance

**Figure 6:**
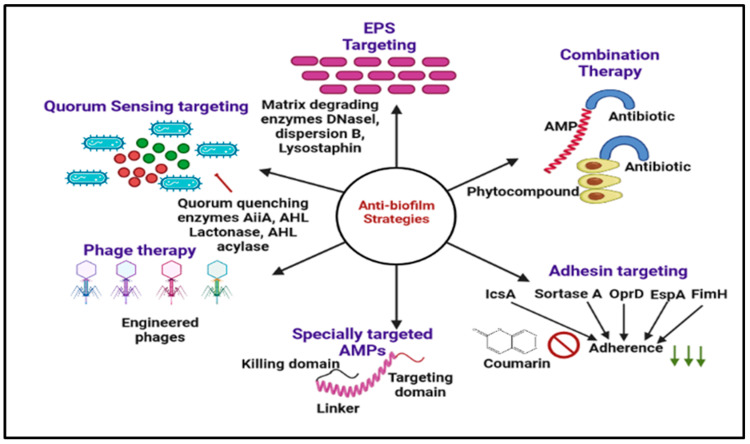
Graphical illustration of the different antibiofilm approaches discussed in this review. EPS targeting (DNaseI, dispersion B, lysostaphin), Phage therapy, nanotechnology, chitosan and derivatives, and antimicrobial peptides.

The increasing case of ‘the non-effectiveness’ of antibiotics while dealing with common microbial infections has opened the door for in-depth study and research on the cause of microbial resistance and dealing with the strategies to combat such resistance. The search for new alternatives to the current conventional therapies should be able to act as broad-spectrum antimicrobial agents while keeping minimal side effects [181]. Some novel methods for combating resistance are the application of chitosan and its derivatives, antimicrobial peptide use of metallic nanoparticles and (QACs) [182], stimuli-responsive materials, and the application of Phage therapy. The details of these new methods are discussed below [183] and summarized in Figure 6.

### 4.1. Use of Chitosan and Its Derivatives

Chitosan is obtained from its natural precursor, chitin, which is commonly found in organisms such as algae, crustaceans, insects, and fungus, etc. When Chitosan undergoes the process of deacetylation either enzymatically or under the effect of an alkaline solution, chitosan formation takes place. Since, chitosan is obtained naturally, it is known for its biocompatibility, biodegradability, non-toxicity, non-immunogenicity, and ease of availability. Owing to its various beneficial properties, several studies from the literature have reported its use as an antioxidant, antimicrobial, and anticancer agent [184]. Chitosan, being a positively charged agent, has been used in various drug delivery systems either as the main component or applied as a coating material to impart a positive charge to the particles. The positive charge on the chitosan has been utilized for electrostatic interaction between surfaces that are either negatively charged and/or have been altered to induce a negative charge on the surface. The prime reason behind using chitosan as an antibiofilm agent is the cellular component of biofilms such as (lipids, exopolysaccharides, and e-DNA), and external components that constitute bacterial membranes such as (lipopolysaccharides or teichoic acids) are all anionic in nature. Thus, positively charged polymeric agents such as chitosan are electrostatically bound to the component at the membrane and disrupt the cellular structure of microbes, leading to the destabilization and leakage of the bacterial membrane. This causes leakage of the internal cellular components, such as proteins and electrolytes, through pore formation, thus completing the bacteriostatic and bactericidal effect [185]. Usually, the science of nanotechnology has been applied in the formulation of chitosan nanoparticles, which have been used for antimicrobial and antibiofilm therapy. With the chitosan nanoparticles being small in size, they can penetrate deep inside the biofilm membrane, thus causing the lysis, disruption, and disaggregation of the biofilm [186,187]. A different group of researchers worked with Chitosan either as a single agent or in combination with other herbal or synthetic molecules with promising antimicrobial effects.

In one such study, *Hongbin* et al. developed a system of minocycline-loaded chitosan and alginate, which reportedly destroyed adherent and planktonic bacterial colonies [188]. *Subhaswaraj* et al. formulated cinnamaldehyde-loaded chitosan NPs, which showed effective inhibition of *P. aeruginosa*-led biofilm formation effecting QS function and altering mortality [189]. In another study, *Jinhong* et al. designed a system in which chitosan and heparin were deposited in alternate manners on poly (ethylene terephthalate) film to carry out an antibacterial effect. A critical outcome of this study was that the system was found to be antiadhesive; hence could be used in implants [190]. Another form of chitosan, quaternized chitosan (QCS), known as HACC (hydroxypropyltrimethyl ammonium chloride chitosan), is antibacterial in nature. It has been widely used in coating implants, tissue engineering, and wound healing. *Peng* et al. conducted their research and suggested that 18% of substituted HACC showed firm antibacterial activity, keeping toxicity to the minimum [49].

### 4.2. Phage Therapy

Phage is the virus that specifically invades and multiplies in bacterial cells. This inherent quality of the virus has been exploited to solve the issue of drug resistance in bacteria due to the biofilm formation. These phages have been explored as they only attack bacterial cells leaving behind healthy host cells and micro flora untouched [191,192]. Since phage therapy involves the steps of virus attachment to the host and transfer of the genetic material into the bacteria, this property was utilized to destroy the extracellular biofilm matrix to cause the bacterial elimination [191]. As the research progressed further, the cocktail of bacteriophage and antibiotics was explored with many studies showing a positive synergistic effect on disrupting biofilms [193]. The group, *Gu* et al., worked on the step-by-step technique for the combined delivery of phages to eradicate *Klebsiella pneumonia* by administering three phages (*GH-K1*, *GH-K2*, and *GH-K3*). The findings showed that when combined phages were delivered, they produced a stronger response against *Klebsiella pneumonia* than individual phages in the murine K7 strain model. They used data to prove their hypothesis that the lowest effective dose of the combined phage level was significantly lower than that of a single phage. Although this idea looks very effective for combating biofilm resistance, much effort still needs to be applied to formulate bacteriophage into some dosage forms. Needs for standardization, optimization, and guarantees of safety and efficacy are some of the questions that need to be addressed before such therapies can be used as a generalized treatment method for the larger population.

## 5. Conclusions and Perspective

Bacterial biofilms are widespread and play a crucial role in antibiotic resistance. Therapy of biofilm-associated infections is nowadays a problematic task for healthcare professionals. There is the urge to develop novel antimicrobial techniques to overcome problems associated with bacterial resistance. As revealed in this review, a vast library of natural resources is available to examine antibiofilm compounds. A number of researches have shown the inhibitory effects of natural drugs on the formation and growth of bacterial biofilms, revealing their potential as substitute treatments for bacterial illnesses. Their possible regulation mechanism was primarily caused by the inhibition of the QS system at different phases of biofilm development. Many plant extracts have also shown potential antibiofilm activity; there molecular structures are not identified, indicating more clinical trials need to be performed. This review also focuses on the quorum quenching (QQ) molecules and natural extracellular polymeric substance inhibitory enzymes and their mechanism of action on biofilm. Most of the natural drugs’ mechanisms are still unknown. More research in this area can overcome drug resistance. Clinical investigations that are now underway mostly concentrate on the external use of oral biofilms formed in dental plaque, periodontitis, and gingivitis. A promising therapy approach for biofilm infections is the combination of antibiotic drugs and herbal antibiofilm drugs for effective remedies. It is vital to conduct in vivo research to assess the dependability of the combined medications because most investigations on these drugs are now conducted in vitro. Based on fingerprint efficacy, QC modelling, and PK-PD (pharmacokinetic–pharmacodynamic) correlation in in vivo investigations, the formation of combined pharmaceutical therapies will be aided by the ability to show the mechanisms of the combined medications.

## Figures and Tables

**Table 2:** List of biosurfactants with their effect on biofilm development.

Class	Microorganism Source	Strains	Effect on Biofilm	Reference
Plantarum MGL-8 (L-MJ)	*Lactobacillus plantarum*	*Staphylococcus aureus*	Controls the genes expression *dltB*, *icaA*, *cidA* responsible for biofilm formation and quorum sensing signalling molecules	[104]
Lipopeptide	*Acinetobacter junii*	*M. canis*	It impacts cell membrane permeability and starts to break down the integrity of the cell membrane	[106]
Rhamnolipids	*Pseudomonas aeruginosa MN1*	*S. aureus*	Intense antiadhesive properties and antibiofilm activity	[108]
Glycolipid	*Burkholderia sp. WYAT7*	*Staphylococcus aureus*	Inhibits the adhesion of planktonic cells and antibiofilm properties.	[109]
Exopolysaccharides	*Pandorea pnomenusa MS5*	*Burkholderia cepacia*	Promotes biofilm destruction	[11]
Glycolipoprotein	*Acinetobacter indicus M6*	*Methicillin resistance strains and Salmonella typhi*	Inhibits EPS synthesis and biofilm formation	[105]
Lipopeptide surfactin-C15	*B. subtilis #309*	*Candida albicans*	Acts on adhesive properties of the cell and shows antibiofilm properties	[107]
Lipopeptide pontifactin	*Pontibacter korlensis SBK-47*	*S. aureus*, *Bacillus subtilis*, *Salmonella typhi*, *and Vibrio cholerae*	Prominent antiadhesive properties	[106]

## Data Availability

Data are included in this paper.
